# Bis(η^7^-cyclo­hepta­trien­yl)tri-μ-hydrido-dimolybdenum(0,I)

**DOI:** 10.1107/S1600536808042244

**Published:** 2008-12-17

**Authors:** Peter G. Jones, Cristian G. Hrib, Sören Randoll, Xian Wu, Matthias Tamm

**Affiliations:** a Institut für Anorganische und Analytische Chemie, Technische Universität Braunschweig, Postfach 3329, 38023 Braunschweig, Germany

## Abstract

In the title compound, [Mo_2_(η^7^-C_7_H_7_)_2_(μ-H)_3_], which displays crystallographic mirror symmetry, two (η^7^-C_7_H_7_)Mo units are linked along the Mo—Mo axis by three bridging hydride ligands. The Mo—Mo distance is 2.5732 (4) Å. The perpendicular distances of the Mo atoms from the C_7_ planes are 1.5827 (8) and 1.5814 (8) Å, with individual Mo—C bond lengths in the range 2.261 (2)–2.2789 (14) Å. Mo—H distances range from 1.77 (3) to 1.85 (4) Å, with Mo—H—Mo angles of 89 (2) and 92 (1)°.

## Related literature

For related literature, see: Alvarez *et al.* (2006 ▶); Darensbourg *et al.* (1980 ▶); Jones *et al.* (1980 ▶); Lin *et al.* (1993 ▶); Süss-Fink & Therrien (2007 ▶); Petersen *et al.* (1981 ▶); Shima & Suzuki (2005 ▶); Tamm *et al.* (2004 ▶, 2006 ▶). 
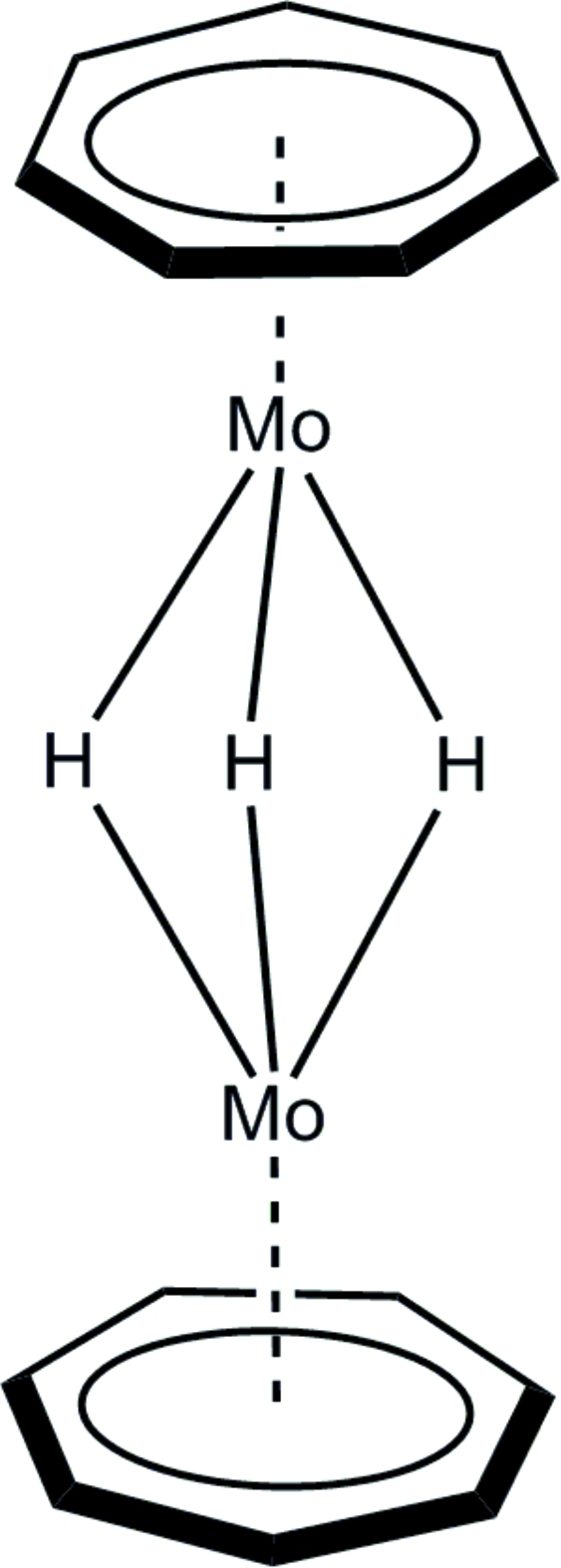


## Experimental

### 

#### Crystal data

[Mo_2_(C_7_H_7_)_2_H_3_]
*M* *_r_* = 377.16
Orthorhombic, 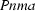

*a* = 17.844 (2) Å
*b* = 11.3036 (16) Å
*c* = 6.2981 (8) Å
*V* = 1270.3 (3) Å^3^
*Z* = 4
Mo *K*α radiation
μ = 1.94 mm^−1^
*T* = 133 (2) K
0.38 × 0.20 × 0.04 mm

#### Data collection

Bruker SMART 1000 CCD diffractometer
Absorption correction: multi-scan (*SADABS*; Bruker, 1998 ▶) *T*_min_ = 0.526, *T*_max_ = 0.926
25354 measured reflections
2023 independent reflections
1871 reflections with *I* > 2σ(*I*)
*R*_int_ = 0.026

#### Refinement

*R*[*F* ^2^ > 2σ(*F* ^2^)] = 0.015
*wR*(*F* ^2^) = 0.041
*S* = 1.06
2023 reflections
86 parameters
H atoms treated by a mixture of independent and constrained refinement
Δρ_max_ = 0.48 e Å^−3^
Δρ_min_ = −0.41 e Å^−3^

### 

Data collection: *SMART* (Bruker, 1998 ▶); cell refinement: *SAINT* (Bruker, 1998 ▶); data reduction: *SAINT*; program(s) used to solve structure: *SHELXS97* (Sheldrick, 2008 ▶); program(s) used to refine structure: *SHELXL97* (Sheldrick, 2008 ▶); molecular graphics: *XP* (Siemens, 1994 ▶); software used to prepare material for publication: *SHELXL97* (Sheldrick, 2008 ▶).

## Supplementary Material

Crystal structure: contains datablocks I, global. DOI: 10.1107/S1600536808042244/bt2832sup1.cif

Structure factors: contains datablocks I. DOI: 10.1107/S1600536808042244/bt2832Isup2.hkl

Additional supplementary materials:  crystallographic information; 3D view; checkCIF report

## Figures and Tables

**Table 1:** Selected geometric parameters (Å, °)

Mo1—C1	2.261 (2)
Mo1—C3	2.2638 (14)
Mo1—C2	2.2740 (15)
Mo1—C4	2.2753 (14)
Mo1—Mo2	2.5732 (4)
Mo1—H9	1.81 (3)
Mo1—H10	1.85 (4)
Mo2—C7	2.2603 (14)
Mo2—C5	2.264 (2)
Mo2—C8	2.2742 (15)
Mo2—C6	2.2789 (14)
Mo2—H9	1.77 (3)
Mo2—H10	1.82 (4)

**Fig. 1.**
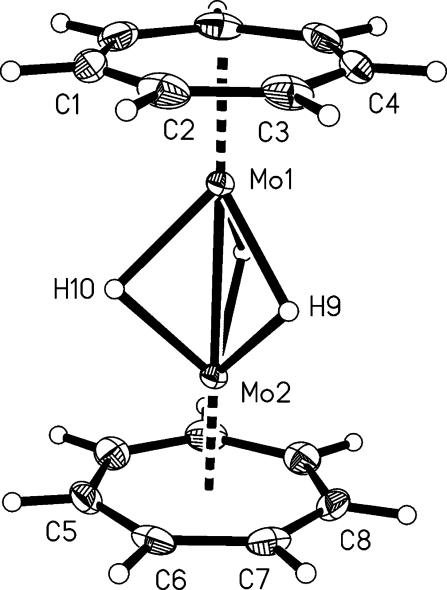
The formula unit of the title compound in the crystal. Ellipsoids represent 50% probability levels.

## supplementary crystallographic information

### Comment

Significant recent attention has been paid to dinuclear trihydrido complexes, which tend to be soluble in both polar organic solvents and water and thus have potential applications in aqueous organometallic chemistry. A useful synthesis of such complexes is reaction of the corresponding aquo complexes with sodium borohydride in water. Here we report the formation of the title compound through the reaction of the corresponding triacetonitrile complex of molybdenum with sodium borohydride in acetonitrile. The same reaction, but in the presence of tricyclohexylphosphane, affords a tetrahydroborate complex (Tamm *et al.*, 2006). Most dinuclear tri- and polyhydrido complexes contain either two cyclopentadienyl rings or two arene rings (Shima *et al.*, 2005; Süss-Fink *et al.*, 2007), and this is thus the first synthesis of a dinuclear trihydrido complex that contains two cycloheptatrienyl rings. If these rings are formally assigned the charge +1, corresponding to an aromatic π system, then the metal oxidation states are mixed (0,I). Related structural motifs have been observed for halide-bridged dimolybdenum complexes (Tamm *et al.*, 2004).

The X-ray structure analysis of the title compound reveals two (η^7^-C_7_H_7_)Mo units linked by three bridging hydrido ligands. The molecule possesses a crystallographic mirror plane passing through both molybdenum atoms, the atoms C1 and H1 of the cycloheptatrienyl ligands, and the hydride H10. The perpendicular distances of the Mo atoms from the C_7_ planes are 1.5827 (8) Å for Mo1 and 1.5814 (8) Å for Mo_2_, with individual Mo—C bond lengths in the range 2.261 (2) – 2.2789 (14) Å. The two C_7_ planes (r.m.s. deviation 0.005, 0.008 Å) are almost parallel, with an interplanar angle of 1.59 (7) Å. The molecular axis, defined as the sequence (Centroid ring 1)—Mo1—Mo2—(Centroid ring 2), is essentially linear, with Cent—Mo—Mo angles of 179°. The Mo—H—Mo bonds can be described as three-centre, two-electron (3c-2 e) bonds, with Mo—H distances between 1.77 (3) and 1.85 (4) Å.

The Mo—Mo bond length of 2.5732 (4) Å is, as expected, shorter than the Mo—Mo bonds in monohydrido (Mo—H—Mo) complexes, where this distance lies in the range 3.4056 (5)–3.540 (1) Å (Petersen *et al.*, 1981; Darensbourg *et al.*, 1980; Lin *et al.*, 1993). The monohydrido complex [Mo_2_(η^5^-C_5_H_5_)_2_(µ-H)(SnPh_3_)(CO)_2_(PCy_2_H)] reported by Alvarez *et al.* (2006) contains a formal Mo≡Mo bond with a length (2.5730 (6) Å) almost identical to that in the title complex. There is only one report of a dihydrido complex with a quadruply bonded Mo—Mo (2.194 (3) Å) unit (Jones *et al.*, 1980).

### Experimental

0.1324 g NaBH_4_ (0.44 mmol) was suspended in ethanol and cooled to 0 °C. A solution of [(η^7^-C_7_H_7_)Mo(NCMe)_3_]PF_6_ (0.1324 g, 3.50 mmol) in acetonitile was added to this suspension. The mixture was stirred at room temperature for several hours. After removal of the solvent and subsequently drying under high vacuum the residue was extracted with hexane/diethyl ether (1:2). Single crystals of the title compound were obtained by cooling of this solution.

### Refinement

The bridging H atoms were identified in difference syntheses and freely refined. Other (aromatic) hydrogen atoms were included using a riding model with C—H 0.95 Å and *U*(H) values fixed at 1.2*U*_iso_(C) of the parent C atom.

### Crystal data

**Table:**

[Mo_2_(C_7_H_7_)_2_H_3_]	*D*_x_ = 1.972 Mg m^−^^3^
*M**_r_* = 377.16	Mo *K*α radiation, λ = 0.71073 Å
Orthorhombic, *P**n**m**a*	Cell parameters from 7837 reflections
*a* = 17.844 (2) Å	θ = 2.3–30.5°
*b* = 11.3036 (16) Å	µ = 1.94 mm^−^^1^
*c* = 6.2981 (8) Å	*T* = 133 K
*V* = 1270.3 (3) Å^3^	Tablet, green
*Z* = 4	0.38 × 0.20 × 0.04 mm
*F*(000) = 740	

### Data collection

**Table:**

Bruker SMART 1000 CCD diffractometer	2023 independent reflections
Radiation source: fine-focus sealed tube	1871 reflections with *I* > 2σ(*I*)
graphite	*R*_int_ = 0.026
Detector resolution: 8.192 pixels mm^-1^	θ_max_ = 30.5°, θ_min_ = 2.3°
ω and φ scans	*h* = −25→25
Absorption correction: multi-scan (*SADABS*; Bruker, 1998)	*k* = −16→15
*T*_min_ = 0.526, *T*_max_ = 0.926	*l* = −8→8
25354 measured reflections	

### Refinement

**Table:**

Refinement on *F*^2^	Primary atom site location: structure-invariant direct methods
Least-squares matrix: full	Secondary atom site location: difference Fourier map
*R*[*F*^2^ > 2σ(*F*^2^)] = 0.015	Hydrogen site location: inferred from neighbouring sites
*wR*(*F*^2^) = 0.041	H atoms treated by a mixture of independent and constrained refinement
*S* = 1.06	*w* = 1/[σ^2^(*F*_o_^2^) + (0.0195*P*)^2^ + 1.0664*P*] where *P* = (*F*_o_^2^ + 2*F*_c_^2^)/3
2023 reflections	(Δ/σ)_max_ = 0.001
86 parameters	Δρ_max_ = 0.48 e Å^−^^3^
0 restraints	Δρ_min_ = −0.41 e Å^−^^3^

### Special details

**Table:**

Geometry. All e.s.d.'s (except the e.s.d. in the dihedral angle between two l.s. planes) are estimated using the full covariance matrix. The cell e.s.d.'s are taken into account individually in the estimation of e.s.d.'s in distances, angles and torsion angles; correlations between e.s.d.'s in cell parameters are only used when they are defined by crystal symmetry. An approximate (isotropic) treatment of cell e.s.d.'s is used for estimating e.s.d.'s involving l.s. planes.
Refinement. Refinement of *F*^2^ against ALL reflections. The weighted *R*-factor *wR* and goodness of fit *S* are based on *F*^2^, conventional *R*-factors *R* are based on *F*, with *F* set to zero for negative *F*^2^. The threshold expression of *F*^2^ > σ(*F*^2^) is used only for calculating *R*-factors(gt) *etc*. and is not relevant to the choice of reflections for refinement. *R*-factors based on *F*^2^ are statistically about twice as large as those based on *F*, and *R*- factors based on ALL data will be even larger.

### Fractional atomic coordinates and isotropic or equivalent isotropic displacement parameters (Å^2^)

**Table:**

	*x*	*y*	*z*	*U*_iso_*/*U*_eq_	
Mo1	0.138195 (8)	0.2500	0.59745 (3)	0.01163 (5)	
Mo2	0.007079 (8)	0.2500	0.42733 (3)	0.01206 (5)	
C1	0.17862 (12)	0.2500	0.9377 (4)	0.0280 (5)	
H1	0.1548	0.2500	1.0725	0.034*	
C2	0.19390 (8)	0.13719 (15)	0.8514 (3)	0.0261 (3)	
H2	0.1804	0.0715	0.9374	0.031*	
C3	0.22664 (8)	0.10988 (14)	0.6537 (3)	0.0247 (3)	
H3	0.2309	0.0280	0.6222	0.030*	
C4	0.25402 (8)	0.18763 (15)	0.4960 (3)	0.0231 (3)	
H4	0.2752	0.1511	0.3741	0.028*	
C5	−0.11081 (11)	0.2500	0.5604 (4)	0.0225 (4)	
H5	−0.1318	0.2500	0.6991	0.027*	
C6	−0.09750 (8)	0.13700 (14)	0.4723 (3)	0.0234 (3)	
H6	−0.1121	0.0714	0.5569	0.028*	
C7	−0.06509 (8)	0.10969 (13)	0.2738 (3)	0.0233 (3)	
H7	−0.0589	0.0278	0.2449	0.028*	
C8	−0.04060 (8)	0.18731 (14)	0.1119 (2)	0.0225 (3)	
H8	−0.0217	0.1507	−0.0129	0.027*	
H9	0.0858 (14)	0.155 (2)	0.421 (4)	0.058 (8)*	
H10	0.041 (2)	0.2500	0.700 (6)	0.061 (11)*	

### Atomic displacement parameters (Å^2^)

**Table:**

	*U*^11^	*U*^22^	*U*^33^	*U*^12^	*U*^13^	*U*^23^
Mo1	0.00941 (7)	0.01301 (8)	0.01246 (8)	0.000	−0.00047 (5)	0.000
Mo2	0.00958 (7)	0.01379 (8)	0.01280 (8)	0.000	−0.00056 (5)	0.000
C1	0.0166 (9)	0.0542 (16)	0.0133 (10)	0.000	−0.0030 (7)	0.000
C2	0.0178 (6)	0.0309 (8)	0.0296 (8)	−0.0025 (6)	−0.0072 (6)	0.0149 (7)
C3	0.0157 (6)	0.0181 (6)	0.0403 (9)	0.0047 (5)	−0.0071 (6)	−0.0001 (6)
C4	0.0119 (6)	0.0330 (8)	0.0243 (8)	0.0045 (5)	0.0004 (5)	−0.0074 (6)
C5	0.0123 (8)	0.0354 (12)	0.0199 (10)	0.000	0.0017 (7)	0.000
C6	0.0141 (6)	0.0272 (7)	0.0289 (8)	−0.0070 (5)	−0.0019 (5)	0.0059 (6)
C7	0.0184 (6)	0.0204 (6)	0.0313 (8)	−0.0036 (5)	−0.0070 (6)	−0.0044 (6)
C8	0.0188 (6)	0.0307 (8)	0.0181 (7)	0.0004 (6)	−0.0045 (5)	−0.0067 (6)

### Geometric parameters (Å, °)

**Table:**

Mo1—C1	2.261 (2)	C1—C2	1.413 (2)
Mo1—C3^i^	2.2638 (14)	C1—C2^i^	1.413 (2)
Mo1—C3	2.2638 (14)	C1—H1	0.9500
Mo1—C2^i^	2.2740 (15)	C2—C3	1.409 (2)
Mo1—C2	2.2740 (15)	C2—H2	0.9500
Mo1—C4^i^	2.2753 (14)	C3—C4	1.414 (2)
Mo1—C4	2.2753 (14)	C3—H3	0.9500
Mo1—Mo2	2.5732 (4)	C4—C4^i^	1.410 (3)
Mo1—H9	1.81 (3)	C4—H4	0.9500
Mo1—H10	1.85 (4)	C5—C6	1.4128 (19)
Mo2—C7	2.2603 (14)	C5—C6^i^	1.4128 (19)
Mo2—C7^i^	2.2603 (14)	C5—H5	0.9500
Mo2—C5	2.264 (2)	C6—C7	1.412 (2)
Mo2—C8	2.2742 (15)	C6—H6	0.9500
Mo2—C8^i^	2.2742 (15)	C7—C8	1.414 (2)
Mo2—C6	2.2789 (14)	C7—H7	0.9500
Mo2—C6^i^	2.2789 (14)	C8—C8^i^	1.417 (3)
Mo2—H9	1.77 (3)	C8—H8	0.9500
Mo2—H10	1.82 (4)		
			
C1—Mo1—C3^i^	68.24 (6)	C8^i^—Mo2—Mo1	134.73 (4)
C1—Mo1—C3	68.24 (6)	C6—Mo2—Mo1	133.85 (4)
C3^i^—Mo1—C3	88.79 (8)	C6^i^—Mo2—Mo1	133.85 (4)
C1—Mo1—C2^i^	36.30 (5)	C7—Mo2—H9	90.9 (8)
C3^i^—Mo1—C2^i^	36.19 (6)	C7^i^—Mo2—H9	150.4 (8)
C3—Mo1—C2^i^	88.71 (6)	C5—Mo2—H9	138.2 (9)
C1—Mo1—C2	36.30 (5)	C8—Mo2—H9	95.0 (8)
C3^i^—Mo1—C2	88.71 (6)	C8^i^—Mo2—H9	117.8 (8)
C3—Mo1—C2	36.19 (6)	C6—Mo2—H9	108.1 (9)
C2^i^—Mo1—C2	68.21 (9)	C6^i^—Mo2—H9	173.4 (8)
C1—Mo1—C4^i^	88.65 (7)	Mo1—Mo2—H9	44.6 (8)
C3^i^—Mo1—C4^i^	36.29 (6)	C7—Mo2—H10	126.4 (6)
C3—Mo1—C4^i^	68.13 (6)	C7^i^—Mo2—H10	126.4 (6)
C2^i^—Mo1—C4^i^	68.09 (6)	C5—Mo2—H10	87.8 (12)
C2—Mo1—C4^i^	88.53 (6)	C8—Mo2—H10	161.5 (2)
C1—Mo1—C4	88.65 (7)	C8^i^—Mo2—H10	161.5 (2)
C3^i^—Mo1—C4	68.13 (6)	C6—Mo2—H10	99.0 (10)
C3—Mo1—C4	36.29 (6)	C6^i^—Mo2—H10	99.0 (10)
C2^i^—Mo1—C4	88.53 (6)	Mo1—Mo2—H10	45.9 (12)
C2—Mo1—C4	68.09 (6)	H9—Mo2—H10	75.9 (12)
C4^i^—Mo1—C4	36.10 (8)	C2—C1—C2^i^	129.0 (2)
C1—Mo1—Mo2	133.21 (6)	C2—C1—Mo1	72.36 (11)
C3^i^—Mo1—Mo2	134.35 (4)	C2^i^—C1—Mo1	72.36 (11)
C3—Mo1—Mo2	134.35 (4)	C2—C1—H1	115.5
C2^i^—Mo1—Mo2	133.65 (4)	C2^i^—C1—H1	115.5
C2—Mo1—Mo2	133.65 (4)	Mo1—C1—H1	134.8
C4^i^—Mo1—Mo2	135.14 (4)	C3—C2—C1	128.13 (16)
C4—Mo1—Mo2	135.14 (4)	C3—C2—Mo1	71.51 (9)
C1—Mo1—H9	138.4 (8)	C1—C2—Mo1	71.34 (11)
C3^i^—Mo1—H9	150.9 (8)	C3—C2—H2	115.9
C3—Mo1—H9	92.2 (8)	C1—C2—H2	115.9
C2^i^—Mo1—H9	172.9 (8)	Mo1—C2—H2	136.7
C2—Mo1—H9	108.9 (8)	C2—C3—C4	128.89 (14)
C4^i^—Mo1—H9	118.8 (8)	C2—C3—Mo1	72.30 (8)
C4—Mo1—H9	96.4 (8)	C4—C3—Mo1	72.30 (8)
Mo2—Mo1—H9	43.5 (8)	C2—C3—H3	115.6
C1—Mo1—H10	88.2 (12)	C4—C3—H3	115.6
C3^i^—Mo1—H10	126.8 (6)	Mo1—C3—H3	134.8
C3—Mo1—H10	126.8 (6)	C4^i^—C4—C3	128.44 (9)
C2^i^—Mo1—H10	99.5 (10)	C4^i^—C4—Mo1	71.95 (4)
C2—Mo1—H10	99.5 (10)	C3—C4—Mo1	71.42 (8)
C4^i^—Mo1—H10	161.7 (2)	C4^i^—C4—H4	115.8
C4—Mo1—H10	161.7 (2)	C3—C4—H4	115.8
Mo2—Mo1—H10	45.0 (12)	Mo1—C4—H4	136.3
H9—Mo1—H10	74.3 (12)	C6—C5—C6^i^	129.4 (2)
C7—Mo2—C7^i^	89.12 (8)	C6—C5—Mo2	72.45 (10)
C7—Mo2—C5	68.23 (5)	C6^i^—C5—Mo2	72.45 (10)
C7^i^—Mo2—C5	68.23 (5)	C6—C5—H5	115.3
C7—Mo2—C8	36.34 (6)	C6^i^—C5—H5	115.3
C7^i^—Mo2—C8	68.40 (6)	Mo2—C5—H5	134.9
C5—Mo2—C8	88.61 (7)	C7—C6—C5	127.92 (15)
C7—Mo2—C8^i^	68.40 (6)	C7—C6—Mo2	71.17 (8)
C7^i^—Mo2—C8^i^	36.34 (6)	C5—C6—Mo2	71.32 (10)
C5—Mo2—C8^i^	88.61 (7)	C7—C6—H6	116.0
C8—Mo2—C8^i^	36.31 (8)	C5—C6—H6	116.0
C7—Mo2—C6	36.23 (6)	Mo2—C6—H6	137.1
C7^i^—Mo2—C6	88.85 (6)	C6—C7—C8	129.00 (14)
C5—Mo2—C6	36.23 (5)	C6—C7—Mo2	72.60 (8)
C8—Mo2—C6	68.13 (6)	C8—C7—Mo2	72.36 (8)
C8^i^—Mo2—C6	88.67 (6)	C6—C7—H7	115.5
C7—Mo2—C6^i^	88.85 (6)	C8—C7—H7	115.5
C7^i^—Mo2—C6^i^	36.23 (6)	Mo2—C7—H7	134.4
C5—Mo2—C6^i^	36.23 (5)	C7—C8—C8^i^	128.35 (9)
C8—Mo2—C6^i^	88.67 (6)	C7—C8—Mo2	71.30 (8)
C8^i^—Mo2—C6^i^	68.13 (6)	C8^i^—C8—Mo2	71.84 (4)
C6—Mo2—C6^i^	68.18 (8)	C7—C8—H8	115.8
C7—Mo2—Mo1	134.12 (4)	C8^i^—C8—H8	115.8
C7^i^—Mo2—Mo1	134.12 (4)	Mo2—C8—H8	136.5
C5—Mo2—Mo1	133.67 (6)	Mo1—H9—Mo2	92 (1)
C8—Mo2—Mo1	134.73 (4)	Mo1—H10—Mo2	89 (2)
			
C1—Mo1—Mo2—C7	−102.21 (6)	C4^i^—Mo1—C3—C2	120.12 (10)
C3^i^—Mo1—Mo2—C7	155.85 (9)	C4—Mo1—C3—C2	142.53 (14)
C3—Mo1—Mo2—C7	−0.26 (9)	Mo2—Mo1—C3—C2	−107.37 (9)
C2^i^—Mo1—Mo2—C7	−153.00 (9)	C1—Mo1—C3—C4	−120.06 (10)
C2—Mo1—Mo2—C7	−51.41 (9)	C3^i^—Mo1—C3—C4	−53.07 (11)
C4^i^—Mo1—Mo2—C7	103.85 (9)	C2^i^—Mo1—C3—C4	−89.27 (10)
C4—Mo1—Mo2—C7	51.74 (9)	C2—Mo1—C3—C4	−142.53 (14)
C1—Mo1—Mo2—C7^i^	102.21 (6)	C4^i^—Mo1—C3—C4	−22.42 (7)
C3^i^—Mo1—Mo2—C7^i^	0.26 (9)	Mo2—Mo1—C3—C4	110.10 (9)
C3—Mo1—Mo2—C7^i^	−155.85 (9)	C2—C3—C4—C4^i^	−1.3 (2)
C2^i^—Mo1—Mo2—C7^i^	51.41 (9)	Mo1—C3—C4—C4^i^	46.82 (7)
C2—Mo1—Mo2—C7^i^	153.00 (9)	C2—C3—C4—Mo1	−48.12 (14)
C4^i^—Mo1—Mo2—C7^i^	−51.74 (9)	C1—Mo1—C4—C4^i^	−89.56 (2)
C4—Mo1—Mo2—C7^i^	−103.85 (9)	C3^i^—Mo1—C4—C4^i^	−22.52 (6)
C1—Mo1—Mo2—C5	0.0	C3—Mo1—C4—C4^i^	−143.08 (8)
C3^i^—Mo1—Mo2—C5	−101.94 (6)	C2^i^—Mo1—C4—C4^i^	−53.25 (5)
C3—Mo1—Mo2—C5	101.94 (6)	C2—Mo1—C4—C4^i^	−120.30 (5)
C2^i^—Mo1—Mo2—C5	−50.79 (7)	Mo2—Mo1—C4—C4^i^	109.12 (5)
C2—Mo1—Mo2—C5	50.79 (7)	C1—Mo1—C4—C3	53.52 (9)
C4^i^—Mo1—Mo2—C5	−153.94 (6)	C3^i^—Mo1—C4—C3	120.56 (13)
C4—Mo1—Mo2—C5	153.94 (6)	C2^i^—Mo1—C4—C3	89.83 (10)
C1—Mo1—Mo2—C8	−153.98 (6)	C2—Mo1—C4—C3	22.78 (9)
C3^i^—Mo1—Mo2—C8	104.07 (9)	C4^i^—Mo1—C4—C3	143.08 (8)
C3—Mo1—Mo2—C8	−52.04 (9)	Mo2—Mo1—C4—C3	−107.80 (9)
C2^i^—Mo1—Mo2—C8	155.22 (9)	C7—Mo2—C5—C6	22.41 (10)
C2—Mo1—Mo2—C8	−103.19 (9)	C7^i^—Mo2—C5—C6	120.56 (12)
C4^i^—Mo1—Mo2—C8	52.08 (9)	C8—Mo2—C5—C6	53.32 (11)
C4—Mo1—Mo2—C8	−0.04 (8)	C8^i^—Mo2—C5—C6	89.65 (11)
C1—Mo1—Mo2—C8^i^	153.98 (6)	C6^i^—Mo2—C5—C6	143.0 (2)
C3^i^—Mo1—Mo2—C8^i^	52.04 (9)	Mo1—Mo2—C5—C6	−108.51 (10)
C3—Mo1—Mo2—C8^i^	−104.07 (9)	C7—Mo2—C5—C6^i^	−120.56 (12)
C2^i^—Mo1—Mo2—C8^i^	103.19 (9)	C7^i^—Mo2—C5—C6^i^	−22.41 (10)
C2—Mo1—Mo2—C8^i^	−155.22 (9)	C8—Mo2—C5—C6^i^	−89.65 (11)
C4^i^—Mo1—Mo2—C8^i^	0.04 (8)	C8^i^—Mo2—C5—C6^i^	−53.32 (11)
C4—Mo1—Mo2—C8^i^	−52.08 (9)	C6—Mo2—C5—C6^i^	−143.0 (2)
C1—Mo1—Mo2—C6	−51.00 (6)	Mo1—Mo2—C5—C6^i^	108.51 (10)
C3^i^—Mo1—Mo2—C6	−152.94 (9)	C6^i^—C5—C6—C7	2.0 (4)
C3—Mo1—Mo2—C6	50.94 (9)	Mo2—C5—C6—C7	−45.95 (16)
C2^i^—Mo1—Mo2—C6	−101.80 (9)	C6^i^—C5—C6—Mo2	48.0 (2)
C2—Mo1—Mo2—C6	−0.21 (9)	C7^i^—Mo2—C6—C7	90.09 (11)
C4^i^—Mo1—Mo2—C6	155.06 (9)	C5—Mo2—C6—C7	143.20 (15)
C4—Mo1—Mo2—C6	102.94 (9)	C8—Mo2—C6—C7	22.96 (9)
C1—Mo1—Mo2—C6^i^	51.00 (6)	C8^i^—Mo2—C6—C7	53.74 (10)
C3^i^—Mo1—Mo2—C6^i^	−50.94 (9)	C6^i^—Mo2—C6—C7	120.65 (8)
C3—Mo1—Mo2—C6^i^	152.94 (9)	Mo1—Mo2—C6—C7	−108.80 (9)
C2^i^—Mo1—Mo2—C6^i^	0.21 (9)	C7—Mo2—C6—C5	−143.20 (15)
C2—Mo1—Mo2—C6^i^	101.80 (9)	C7^i^—Mo2—C6—C5	−53.11 (11)
C4^i^—Mo1—Mo2—C6^i^	−102.94 (9)	C8—Mo2—C6—C5	−120.24 (12)
C4—Mo1—Mo2—C6^i^	−155.06 (9)	C8^i^—Mo2—C6—C5	−89.46 (11)
C3^i^—Mo1—C1—C2	−120.18 (12)	C6^i^—Mo2—C6—C5	−22.55 (12)
C3—Mo1—C1—C2	−22.42 (10)	Mo1—Mo2—C6—C5	108.00 (11)
C2^i^—Mo1—C1—C2	−142.6 (2)	C5—C6—C7—C8	−2.5 (3)
C4^i^—Mo1—C1—C2	−89.35 (11)	Mo2—C6—C7—C8	−48.52 (14)
C4—Mo1—C1—C2	−53.24 (11)	C5—C6—C7—Mo2	46.00 (16)
Mo2—Mo1—C1—C2	108.70 (10)	C7^i^—Mo2—C7—C6	−89.25 (10)
C3^i^—Mo1—C1—C2^i^	22.42 (10)	C5—Mo2—C7—C6	−22.41 (9)
C3—Mo1—C1—C2^i^	120.18 (12)	C8—Mo2—C7—C6	−142.34 (13)
C2—Mo1—C1—C2^i^	142.6 (2)	C8^i^—Mo2—C7—C6	−119.89 (10)
C4^i^—Mo1—C1—C2^i^	53.24 (11)	C6^i^—Mo2—C7—C6	−53.01 (10)
C4—Mo1—C1—C2^i^	89.35 (11)	Mo1—Mo2—C7—C6	108.01 (9)
Mo2—Mo1—C1—C2^i^	−108.70 (10)	C7^i^—Mo2—C7—C8	53.09 (10)
C2^i^—C1—C2—C3	−1.8 (4)	C5—Mo2—C7—C8	119.93 (10)
Mo1—C1—C2—C3	46.32 (16)	C8^i^—Mo2—C7—C8	22.45 (7)
C2^i^—C1—C2—Mo1	−48.2 (2)	C6—Mo2—C7—C8	142.34 (13)
C1—Mo1—C2—C3	−143.14 (15)	C6^i^—Mo2—C7—C8	89.33 (10)
C3^i^—Mo1—C2—C3	−89.72 (11)	Mo1—Mo2—C7—C8	−109.65 (9)
C2^i^—Mo1—C2—C3	−120.36 (8)	C6—C7—C8—C8^i^	2.0 (2)
C4^i^—Mo1—C2—C3	−53.42 (10)	Mo2—C7—C8—C8^i^	−46.58 (7)
C4—Mo1—C2—C3	−22.83 (9)	C6—C7—C8—Mo2	48.61 (14)
Mo2—Mo1—C2—C3	109.41 (8)	C7^i^—Mo2—C8—C7	−120.70 (12)
C3^i^—Mo1—C2—C1	53.42 (11)	C5—Mo2—C8—C7	−53.62 (8)
C3—Mo1—C2—C1	143.14 (15)	C8^i^—Mo2—C8—C7	−143.16 (8)
C2^i^—Mo1—C2—C1	22.78 (12)	C6—Mo2—C8—C7	−22.90 (9)
C4^i^—Mo1—C2—C1	89.72 (11)	C6^i^—Mo2—C8—C7	−89.86 (10)
C4—Mo1—C2—C1	120.31 (12)	Mo1—Mo2—C8—C7	107.88 (8)
Mo2—Mo1—C2—C1	−107.45 (11)	C7—Mo2—C8—C8^i^	143.16 (8)
C1—C2—C3—C4	1.9 (3)	C7^i^—Mo2—C8—C8^i^	22.47 (5)
Mo1—C2—C3—C4	48.12 (14)	C5—Mo2—C8—C8^i^	89.54 (2)
C1—C2—C3—Mo1	−46.26 (17)	C6—Mo2—C8—C8^i^	120.26 (5)
C1—Mo1—C3—C2	22.48 (9)	C6^i^—Mo2—C8—C8^i^	53.30 (5)
C3^i^—Mo1—C3—C2	89.47 (10)	Mo1—Mo2—C8—C8^i^	−108.96 (5)
C2^i^—Mo1—C3—C2	53.27 (10)		

Symmetry codes: (i) *x*, −*y*+1/2, *z*.
